# Bushes in the Tree of Life

**DOI:** 10.1371/journal.pbio.0040352

**Published:** 2006-11-14

**Authors:** Antonis Rokas, Sean B Carroll

## Abstract

A discussion of how homoplasy (the frequency of independently evolved characters) and the spacing of cladogenetic events limit our ability to reconstruct the tree of life using existing phylogenetic methods.

Genome analyses are delivering unprecedented amounts of data from an abundance of organisms, raising expectations that in the near future, resolving the tree of life (TOL) will simply be a matter of data collection. However, recent analyses of some key clades in life's history have produced bushes and not resolved trees. The patterns observed in these clades are both important signals of biological history and symptoms of fundamental challenges that must be confronted. Here we examine how the combination of the spacing of cladogenetic events and the high frequency of independently evolved characters (homoplasy) limit the resolution of ancient divergences. Because some histories may not be resolvable by even vast increases in amounts of conventional data, the identification of new molecular characters will be crucial to future progress.

“… there is, after all, one true tree of life, the unique pattern of evolutionary branchings that actually happened. It exists. It is in principle knowable. We don't know it all yet. By 2050 we should – or if we do not, we shall have been defeated only at the terminal twigs, by the sheer number of species.”

    
*Richard Dawkins [1]*

Who are tetrapods' closest living relatives? Which is the earliest-branching animal phylum? Answers to such fundamental questions would be easy if the historical connections among all living organisms in the TOL were known. Obtaining an accurate depiction of the evolutionary history of all living organisms has been and remains one of biology's great challenges.

The discipline primarily responsible for assembling the TOL—molecular systematics—has produced many new insights by illuminating episodes in life's history, posing new hypotheses, as well as providing the evolutionary framework within which new discoveries can be interpreted [2]. Molecular systematics has surmounted the confusion stemming from comparisons of morphologically disparate species to reveal unexpected evolutionary relationships such as the Afrotheria, a clade composed of strikingly different mammals including elephants, aardvarks, manatees, and golden moles [3]. It has also aided the placement of the history of life in a temporal framework, shedding light on key evolutionary events independently of—and in many cases well before—the availability of fossil or biogeographic evidence. A notable example is the discovery that the Hawaiian drosophilid lineage predates by many million years the oldest extant Hawaiian island, having originated on islands now submerged [4].

With such powers in mind, for the casual reader of the phylogenetics literature, the contents table of the May 2005 issue of *Molecular Biology and Evolution* may be somewhat bewildering. Two articles only a few pages apart paradoxically provide evidence for both rejecting [5] and corroborating [6] the existence of Ecdysozoa, a metazoan clade uniting moulting phyla such as arthropods and nematodes. Surely, (at least) one of these studies must be wrong; and yet, identifying which is not as straightforward as one might think. Cases like the Ecdysozoa are a common sight in the molecular systematics literature [2,3,7–12]. How can it be that despite the availability of large amounts of data and powerful statistical techniques, evolutionary trees upon which experts agree have not been reached?

Here we discuss how and why certain critical parts of the TOL may be difficult to resolve, regardless of the quantity of conventional data available. We do not mean this essay to be a comprehensive review of molecular systematics. Rather, we have focused on the emerging evidence from genome-scale studies on several branches of the TOL that sharply contrasts with viewpoints—such as that in the opening quotation—which imply that the assembly of all branches of the TOL will simply be a matter of data collection. We view this difficulty in obtaining full resolution of particular clades—when given substantial data—as both biologically informative and a pressing methodological challenge. The recurring discovery of persistently unresolved clades (bushes) should force a re-evaluation of several widely held assumptions of molecular systematics. Now, as the field is transformed from a data-limited to an analysis-limited discipline, it is an opportune time to do so.

## Stems and Branches: Trees and Bushes

The TOL has been molded by cladogenesis and extinction. Starting from a single lineage that undergoes cladogenesis and splits into two, the rate at which the lineages arising from this cladogenetic event undergo further cladogenetic events determines the lengths of the nascent stems. Once these stems have been generated, the only process that can modify their lengths is extinction. At its core, the elucidation of evolutionary relationships is the identification, through statistical means, of the tree's stems.

It is vital to appreciate that cladogenetic events typically begin as inconspicuous divergences between very similar populations. The subsequent divergences in phenotypic appearances are not phylogenetically informative. This is especially important to bear in mind for extant representatives of clades (Box 1) that originated hundreds of million years ago, in deep time. These forms represent the end products of long series of evolutionary changes [13]. The features by which we recognize these clades today have succeeded the cladogenetic events we are trying to disentangle; their current divergence in body-plan architecture will be uninformative as to the time spans and branching order of the stems separating these clades.

**Figure 1:**
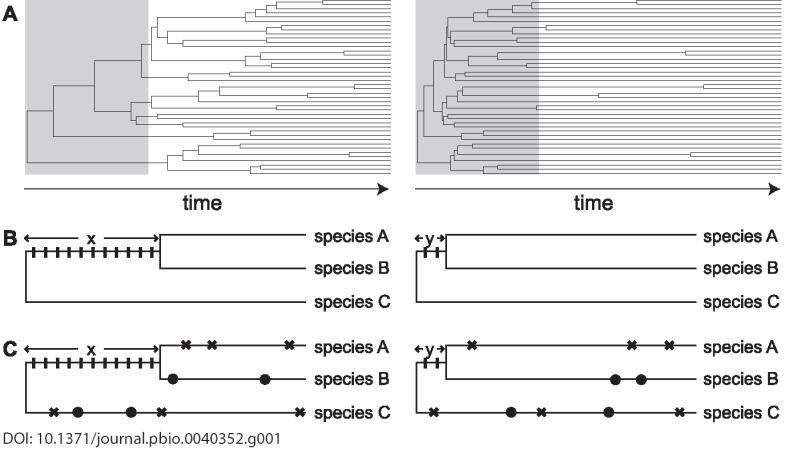
The Shape of a Clade Influences its Resolvability (A) Early in a clade's history (gray box), the number of cladogenetic events is smaller and the length of stems larger in tree-like (left) relative to bush-like clades (right). (B) In the absence of homoplasy, the number of PICs for a stem is proportional to its time span; many PICs (rectangles) accumulated on the long stem *x* (left), whereas few PICs accumulated on the short stem *y* (right). (C) When the stem time span is long, the effect of homoplastic characters (crosses supporting a clade of species A and C and bullets supporting a clade of species B and C) is not sufficient to obscure the true signal (left). In contrast, the same number of homoplastic characters is sufficient to mislead reconstruction of short stems (right), because the number of homoplastic characters shared between species A and C (three crosses in each of the two species) is larger than the number of true PICs (two rectangles).

In the course of evolution, the relative rates of cladogenesis and extinction have differed enormously across clades [14], resulting in different tree shapes (Figure 1A). For example, the occurrence of cladogenetic events at widely spaced intervals generates clades characterized by long stems, and as time elapses, the phylogeny acquires a tree-like shape. In contrast, a radiation where a series of cladogenetic events occurs within a short time span generates a clade characterized by short stems. As the elapsed time since the radiation increases, the external branches lengthen and the phylogeny becomes bush-like.

The relative shape of clades is a key determinant of the prospects for the accurate reconstruction of their history [15]. This is because the amount of signal for a given stem is finite and proportional to the time span of the stem in question [16]. In a parsimony framework—which we illustrate here for simplicity—the signal for a given stem essentially equals the number of parsimony-informative characters (PICs; Box 1) supporting that stem (Figure 1B).

Because molecular characters typically have a few alternative states, the probability of several species acquiring the same nucleotide or amino acid independently (homoplasy; Box 1) is significant and can overwhelm the true historical signal given sufficient time, irrespective of the phylogenetic method used [17]. Bush-shaped clades are characterized by longer external branches relative to the stems, and therefore more homoplastic changes are likely to occur on the external branches [18], thus generating characters that conflict with the true phylogenetic signal (Figure 1C).

One strategy to circumvent homoplasy has been the use of rare genomic changes (RGCs; Box 1). RGCs have more alternative states and thus are less vulnerable to homoplasy. Their solid support for a clade of cetaceans (whales and dolphins) and hippopotamuses within cetartiodactyls is a stellar example of their power [19]. However, two caveats are worth mentioning in the use of all characters (RGCs as well as linear sequence data) for phylogenetic reconstruction purposes. First, all characters can be subject to horizontal gene transfer [20,21] (Box 1), which obscures organismic phylogenetic history. Second, when stems are short in absolute time span, characters can be influenced by population-level processes, such as the lineage sorting of ancestral polymorphisms [22] and hybridization [23] (Box 1). In all such cases, there is not a single true molecular phylogeny, because the species' DNA record is an amalgam of different evolutionary histories.

Thus, absolutely or relatively short stems present distinct challenges that could be described as the bane of the molecular systematist. Yet, it is precisely these stems—associated with some of the most interesting episodes in life's history—that most intrigue the evolutionist. Analyses of large molecular datasets from clades at different time depths of the TOL illustrate how short stems, whether placed just 6 million or 600 million years in the past, can confound phylogenetic resolution. Below, we describe four exemplar stems and dissect the major factors hindering phylogenetic resolution.

## Bushes in the Tree of Life

**Figure 2:**
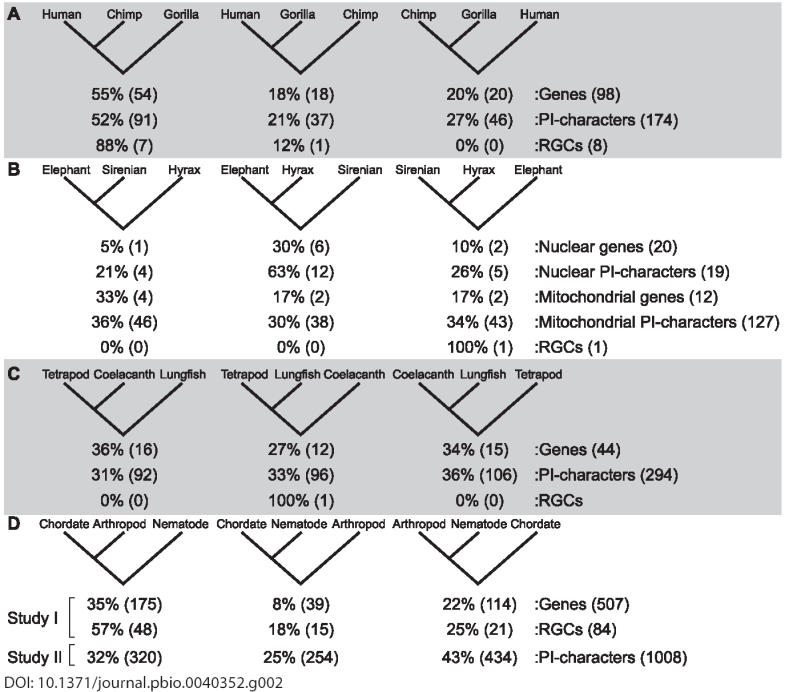
Four Notable Bushes at Different Temporal Depths of the TOL (A) The human/chimpanzee/gorilla tree (5–8 million years ago). (B) The elephant/sirenian/hyrax bush (57–65 million years ago). (C) The tetrapod/coelacanth/lungfish bush (370–390 million years ago). (D) The metazoan superbush (>550 million years ago). In each panel, the three alternative topologies for each set of taxa are shown. Below each topology, the percentage and number (in parentheses) of genes, PICs, and RGCs supporting that topology are shown (when available). Numbers of genes supporting each topology in (A), (C), and (D) are based on maximum likelihood analyses; numbers in (B) are based on parsimony. The observed conflicts are not dependent on the optimality criterion used; similar results were obtained by analyses of the data under a variety of widely used optimality criteria (see references below). A fraction of genes in each panel is uninformative: (A), 6 of 98 genes; (B), 9 of 20 nuclear genes; (C), 1 of 44 genes; and (D), 179 of 507 genes. The single-codon indel supporting the tetrapod/lungfish topology [69] could be homoplastic or even the result of lineage sorting (C). Data for each panel are from the following: (A), [24–27]; (B), [3,30,31]; (C), [8,69]; and (D), [9,10].

**The gorilla/chimp/human tree (5–8 million years ago).** Whereas genomic analyses have shown that at the species level, chimpanzees are humans' closest relatives [24], many of the genes and genomic segments examined have followed different evolutionary paths [24–26]. Specifically, analyses of almost 100 genes (under two different optimality criteria) show that ~55% of genes support a human-chimpanzee clade, 40% are evenly split among the two alternative topologies, with the remaining genes being uninformative [25,26] (Figure 2A). Similarly, whereas 76% of PICs from a genome-scale survey support a human–chimpanzee clade, 24% of PICs disagree [24] (Figure 2A).

What can account for this conflict in such a recent clade? The short stem (~2 million years) leading to the human–chimpanzee clade strongly suggests that the culprit is lineage sorting [24,26]. The number of homoplastic characters are also surprising for a young clade, accounting for up to 32% of the conflict present in the PICs [24]. Transposon-insertion RGCs also offer support for the human–chimpanzee clade [27] (Figure 2A), but even these data include one character that conflicts with the species tree—yet another indicator of lineage sorting. And this may be too simplistic a view of how humans split from their primate relatives; the spatial distribution of genetic variation in primate genomes has raised the possibility of hybridization between the human and chimp lineages [24].

The phylogenetic patterns observed in these primates are by no means a unique circumstance on the TOL. Clades of similar age also exhibit multiple gene genealogies [28,29]. Given the complexity of the cladogenetic process revealed by the study of these young clades and the difficulties encountered in reconstructing their history, one can begin to anticipate the challenge of resolving clades with similar short stems but that originated deeper in time.

**The elephant/sirenian/hyrax bush (57–65 million years ago).** The relationships among elephants, sirenians, and hyraxes are uncertain, despite the availability of substantial amounts and kinds of molecular data [3] (Figure 2B). Data from 20 nuclear genes have failed to resolve this stem [3,30], because only a handful of PICs are available to weigh on the problem [3] (Figure 2B). Most other mammalian stems at similar evolutionary depths are supported by many more PICs. Furthermore, only a single RGC has been identified for this stem [3] —again contrasting with the many RGCs identified for other stems at similar evolutionary depths. Crucially, the phylogeny supported by nuclear PICs [30] conflicts with the phylogeny supported by the single RGC [3], which in turn conflicts with the phylogeny supported by mitochondrial PICs [31] (Figure 2B). The DNA record suggests that the three lineages split off from each other in quick succession, geologically speaking, but the phylogenetic relationships among the three orders cannot be reached at present.

**The coelacanth/lungfish/tetrapod bush (370–390 million years ago).** The cladogenetic events that gave rise to the tetrapod, coelacanth, and lungfish lineages have also proven difficult to resolve. The analysis of 44 genes (under three different optimality criteria) and the approximately 300 PICs found therein equally support each of the three alternative phylogenies [8] (Figure 2C). The lack of resolution is again suggestive of a short stem, a finding consistent with fossil evidence indicating that this stem is unlikely to have been longer than approximately 20 million years [32]. The even distribution of the PICs across the three alternative phylogenies [8] is explained by the even spread of homoplasy across the three long external branches leading to tetrapods, coelacanths, and lungfish. Indeed, this pattern of distribution of PICs is diagnostic of bushy clades [33]. Despite more than a dozen molecular phylogenetic analyses over the last 15 years and the current availability of an abundance of molecular sequence data, our knowledge as to the closest living relative of tetrapods is still uncertain.

**The metazoan superbush (>550 million years ago).** A similar inability of still larger datasets to resolve cladogenetic patterns is observed among metazoan clades that diverged even farther back in time. Many recent studies have reported support for many alternative conflicting phylogenies [5,6,9,10]. For example, Wolf and colleagues [9] analyzed 507 genes by maximum likelihood, finding support for Coelomata—a clade that joins phyla possessing a true coelom, such as arthropods and chordates, to the exclusion of phyla without one, such as nematodes (left-most tree in Figure 2D). In contrast, Dopazo and Dopazo [10] analyzed 610 genes also by maximum likelihood and, after exclusion of genes evolving at a faster rate in nematodes, found support for Ecdysozoa (rightmost tree in Figure 2D).

Three observations generally hold true across metazoan datasets that indicate the pervasive influence of homoplasy at these evolutionary depths. First, a large fraction of single genes produce phylogenies of poor quality. For example, Wolf and colleagues [9] omitted 35% of single genes from their data matrix, because those genes produced phylogenies at odds with conventional wisdom (Figure 2D). Second, in all studies, a large fraction of characters—genes, PICs or RGCs—disagree with the optimal phylogeny, indicating the existence of serious conflict in the DNA record. For example, the majority of PICs conflict with the optimal topology in the Dopazo and Dopazo study [10]. Third, the conflict among these and other studies in metazoan phylogenetics [11,12] is occurring at very “high” taxonomic levels—above or at the phylum level.

The problems illustrated by these four clades are representative of those encountered at a variety of time depths across the TOL [2,7,11,12,33]. What is exceptional about these clades is that they have received the greatest data collection efforts and analysis. The persistent resolution of problems in the face of (a) increasing amounts and different kinds of data and (b) state-of-the-art analytical methodology suggest that other less–well analyzed, absolutely or relatively short stems in the TOL may pose similar challenges and be refractory to resolution with comparable datasets.

## Why Hundreds of Genes Might Not Suffice

**Excess homoplasy and the limits of phylogenetic resolution.** Analyses of the four exemplar stems point to homoplasy as a major contributor to the observed lack of resolution. Homoplasy has long been appreciated in theoretical phylogenetics, with much effort invested into understanding its causes and providing corrections for them [18]. However, the observed patterns (Figure 2) give cause for concern that the extent of homoplasy is much greater than expected under widely accepted models of sequence evolution and that the attendant consequences for the limits to phylogenetic resolution are not sufficiently appreciated.

For instance, theory [34] and simulation analyses [8] predict that a small fraction of substitutions will be homoplastic by chance (about 2–5%, depending upon model assumptions and evolutionary distances). However, analysis of the elephant/sirenian/hyrax dataset and the coelacanth/lungfish/ tetrapod dataset indicates that the actual level of homoplasy is ~10% of amino acid substitutions in the first case (178 homoplastic/1,743 total substitutions) and ~15% in the second case (588 homoplastic/3,800 total substitutions), several times greater than expected [8,34]. Similar high levels of homoplasy exist in datasets from other bushy clades [35] (unpublished data) and hold irrespective of analytical methodology [8].

Many processes bias molecular evolution—such as deviation in amino acid composition [36,37], unequal rates of evolution across sites [38] or lineages [39], nonindependent substitutions [40] and selection [41]—and increase levels of homoplasy and compound the challenge of accurate reconstruction [42]. Although we may be uncertain at present as to the causes of homoplasy, there are substantial grounds for considering the role of selection [41]. Purifying selection has been shown to constrain what changes are permitted at variable sites [36,43]. Furthermore, recent studies indicate that a significant fraction of genes [44,45], including many genes commonly used for molecular systematics [36,43,46–48], has been shaped by positive selection, accounting for perhaps 35–45% of all amino acid substitutions [44]. The high levels of homoplasy observed may be the outcome of the action of selection on the proteome [36,47,49].

No matter what the causes, the consequence of greater-than-expected levels of homoplasy is the imposition of even greater limits on the resolution of clades in deep time. Homoplasy on the external branches can swamp the signal on the stems [18]. For example, if only ~5% of substitutions are homoplastic, then a practical limit to stem resolution is reached when the ratio of external branch to stem length exceeds 20:1. Although the effect of homoplasy on phylogenetic reconstruction may be reduced by the addition of taxa [50,51], this is not always so [52–54]. Perhaps more importantly, several lineages exist for which no additional species can be sampled (Figure 2B and 2C). Thus, the accurate resolution of a <20-million-year-long stem in a 400-million-year-old clade (Figure 2C) or a <30-million-year-long stem in a 600-million-year-old clade (Figure 2D) may not be possible with current practices [33,55].

**Barking up the wrong trees: Systematic bias in large datasets.** A second major consequence of homoplasy is the risk of systematic bias in large dataset analyses. Specifically, long external branches typically harbor high levels of homoplasy, which can positively mislead phylogenetic inference [39], leading to the well-known phenomenon of long-branch attraction (Box 1). Therefore, when levels of homoplasy are high, caution must be used in interpreting high clade-support values. For example, in the case of metazoan superclades (Figure 2D) what has been reported in two different studies is not a lack of resolution but two apparently well supported but contradicting phylogenies.

A simple numerical example illustrates the issue. Consider a dataset in which 53 PICs support one phylogeny—call it phylogeny A—and 47 PICs support phylogeny B, which is in conflict with phylogeny A. After crunching the numbers, it can be shown that phylogeny A will be supported by a bootstrap value of ~72%. Now consider what happens to clade support if the character set is expanded but the proportion of PICs supporting each phylogeny remains the same. With 530 PICs supporting phylogeny A and 470 PICs supporting phylogeny B, the bootstrap value obtained in support of phylogeny A will increase to ~97%. Thus, given that investigations of metazoan clades use genome-scale datasets, the recovery of 100% support is not surprising. However, although it is natural to place confidence in such high support values, one must be wary when the number of homoplastic characters is high. Small differences between study designs—such as in dataset construction and the selection of characters or genes analyzed—skew the distribution of PICs and produce the observed absolute support for conflicting clade phylogenies [5,6,9–12]. Thus, a priori expectations of obtaining fully resolved topologies [56] combined with the use of large amounts of data (which generate high support values) can make trees out of bushes.

**Box 1. Glossary**

**Clade:** A group of organisms is considered a clade when it includes all and only all of the descendants arising from a most recent common ancestor.

**Homoplasy:** Shared characters found in different branches of a phylogenetic tree not directly inherited from a common ancestor; these may arise by chance or selection.

**Horizontal gene transfer:** The occurrence of transfer of genes between genetically isolated populations or species [20]. Gene transfer obscures the evolutionary history of organisms, because the phylogenies of genes that have undergone transfer differ from the overlying species phylogeny.

**Hybridization:** The occurrence of gene flow between genetically isolated populations [23].

**Lineage sorting:** The process by which incomplete sorting of ancestrally polymorphic alleles of molecular characters leads to character histories differing from the species' history. Lineage sorting typically occurs in stems spanning less than 2–3 million years, the exact time span being determined by population size and generation time.

**Long-branch attraction:** When the branches leading to certain species are very long, the rate of occurrence of parallel and convergent substitutions at these long branches can become sufficiently high and overwhelm the true historical signal at the stems [18].

**Parsimony-informative characters (PICs):** Those characters in a dataset that have two or more states that are each present in more than one species in the dataset. In a parsimony framework, the distribution of PICs determines the optimal phylogeny.

**Rare genomic changes (RGCs):** Rare mutational events—such as retroposon integrations [3], insertions and deletions in coding sequences [69], and gains and losses of introns [9]—that generally exhibit lower levels of homoplasy, because they are less likely to occur in the same precise way independently [62].

## What Will it Take to See the Trees?

Can we realistically hope to resolve diversification events spanning a few or even tens of millions of years that occurred in deep time? It is widely accepted that nucleotide data are of limited use for resolving deep divergences because of mutational saturation and homoplasy [57]. Until the recent expansion in available data, it has not been possible to fully explore what the limits of the protein record might be. Like others in the field [5,8,9], we also had expectations that scaling up dataset size would be sufficient to resolve interesting groups [29,33]. The evidence presented here suggests that large amounts of conventional characters will not always suffice, even if analyzed by state-of-the-art methodology. Just as it would be futile to use radioisotopes with modest half lives to date ancient rocks, it appears unrealistic to expect conventional linear, homoplasy-sensitive sequences to reliably resolve series of events that transpired in a small fraction of deep time. Although we have known this from theory [58], we are now confronted with the actual pattern of molecular evolution.

We see two urgent priorities for the endeavour to assemble the TOL to succeed. First, the prevalence and causes of homoplasy need to be better understood so that improved models of molecular evolution that account for the noise in the protein record may be developed. It is perhaps indicative of the degree of difficulty involved in reconciling observed patterns in the molecular record with theoretical expectations that the area of theoretical phylogenetics is one in which much effort and progress has been made in recent years [18,59–61]. Second, molecular systematics must now move beyond conventional characters and mine genomic data for new, less-homoplastic characters such as RGCs [62].

## What's Wrong with Bushes?

The identification of clades is of fundamental importance to molecular systematics [63]. It is perhaps for this reason that over the years, systematists have emphasized reconstructing the topology of trees, while placing much less emphasis on the temporal information conveyed by unresolved stems. Currently, phylogenetic bushes are considered experimental failures. But that is seeing the glass as half empty. A bush in which series of cladogenetic events lie crammed and unresolved within a small section of a larger tree does harbour historical information [33,56]. Although it may be heresy to say so, it could be argued that knowing that strikingly different groups form a clade and that the time spans between the branching of these groups must have been very short, makes the knowledge of the branching order among groups potentially a secondary concern.

For example, the lack of phylogenetic resolution at the base of the tetrapod/lungfish/coelacanth clade has not hampered in the least evolutionary research on the anatomical changes that occurred early on in the evolution of the tetrapod lineage [64,65]. Similarly, if the origin of most bilaterian phyla was compressed in time [33], more than 550 million years later it may matter little to know the exact relationships between most phyla to understand the evolution of the molecular tool kit that enabled the evolution of the body plans of the 35 or so animal phyla [66–68].

We submit that if the current efforts to assemble the TOL have, by 2050 (if not much sooner), assembled an arborescent bush of life, Dawkins' prediction will have come to fruition.

## Abbreviations

TOL: tree of life
PIC: parsimony-informative character
RGC: rare genomic change
